# Arginine deiminase pathway is far more important than urease for acid resistance and intracellular survival in *Laribacter hongkongensis*: a possible result of *arc* gene cassette duplication

**DOI:** 10.1186/1471-2180-14-42

**Published:** 2014-02-17

**Authors:** Lifeng Xiong, Jade LL Teng, Rory M Watt, Biao Kan, Susanna KP Lau, Patrick CY Woo

**Affiliations:** 1 Department of Microbiology, Queen Mary Hospital, The University of Hong Kong, University Pathology Building, Hong Kong, China; 2 Research Centre of Infection and Immunology, The University of Hong Kong, Hong Kong, China; 3 Oral Biosciences, The University of Hong Kong, Hong Kong, China; 4 State Key Laboratory for Infectious Disease Prevention and Control, National Institute for Communicable Disease Control and Prevention, Beijing, China; 5 State Key Laboratory of Emerging Infectious Diseases, Hong Kong, China; 6 Carol Yu Centre for Infection, The University of Hong Kong, Hong Kong, China

**Keywords:** *Laribacter hongkongensis*, Acid resistance, Arginine deiminase pathway, Microbe-host interaction

## Abstract

**Background:**

*Laribacter hongkongensis* is a Gram-negative, urease-positive bacillus associated with invasive bacteremic infections in liver cirrhosis patients and fish-borne community-acquired gastroenteritis and traveler’s diarrhea. Its mechanisms of adaptation to various environmental niches and host defense evasion are largely unknown. During the process of analyzing the *L. hongkongensis* genome, a complete urease cassette and two adjacent *arc* gene cassettes were found. We hypothesize that the urease cassette and/or the *arc* gene cassettes are important for *L. hongkongensis* to survive in acidic environment and macrophages. In this study, we tested this hypothesis by constructing single, double and triple non-polar deletion mutants of the urease and two *arc* gene cassettes of *L. hongkongensis* using the conjugation-mediated gene deletion system and examining their effects in acidic environment *in vitro*, in macrophages and in a mouse model.

**Results:**

HLHK9∆*ureA*, HLHK9∆*ureC*, HLHK9∆*ureD* and HLHK9∆*ureE* all exhibited no urease activity. HLHK9∆*arcA1* and HLHK9∆*arcA2* both exhibited arginine deiminase (ADI) activities, but HLHK9∆*arcA1/arcA2* double deletion mutant exhibited no ADI activity. At pH 2 and 3, survival of HLHK9∆*arcA1/arcA2* and HLHK9∆*ureA/arcA1/arcA2* were markedly decreased (p < 0.001) but that of HLHK9∆*ureA* was slightly decreased (p < 0.05), compared to wild type *L. hongkongensis* HLHK9. Survival of HLHK9∆*ureA/arcA1/arcA2* and HLHK9∆*arcA1/arcA2* in macrophages were also markedly decreased (p < 0.001 and p < 0.01 respectively) but that of HLHK9∆*ureA* was slightly decreased (p < 0.05), compared to HLHK9, although expression of *arcA1*, *arcA2* and *ureA* genes were all upregulated. Using a mouse model, HLHK9∆*ureA* exhibited similar survival compared to HLHK9 after passing through the murine stomach, but survival of HLHK9∆*arcA1/arcA2* and HLHK9∆*ureA/arcA1/arcA2* were markedly reduced (p < 0.01).

**Conclusions:**

In contrast to other important gastrointestinal tract pathogens, ADI pathway is far more important than urease for acid resistance and intracellular survival in *L. hongkongensis*. The gene duplication of the *arc* gene cassettes could be a result of their functional importance in *L. hongkongensis*.

## Background

*Laribacter hongkongensis* is a Gram-negative, facultative anaerobic, motile, S-shaped, asaccharolytic, urease-positive bacillus that belongs to the *Neisseriaceae* family of β-proteobacteria [1]. It was first isolated from the blood and thoracic empyema of an alcoholic liver cirrhosis patient in Hong Kong [1]. Recently, it was also recovered from the blood culture of a Korean patient with liver cirrhosis as a result of Wilson’s disease [2]. These cases make chronic liver disease a distinct possible risk factor for invasive *L. hongkongensis* infections, where intestinal mucosal edema and local immunosuppression secondary to portal venous congestion vasculopathy due to liver cirrhosis predisposed the patients to *L. hongkongensis* invasion through the gastrointestinal mucosa. In addition to invasive bacteremic infections, *L. hongkongensis* is also associated with community-acquired gastroenteritis and traveler’s diarrhea [3]. *L. hongkongensis* is likely to be globally distributed, as travel histories from patients suggested its presence in at least four continents: Asia, Europe, Africa and Central America [3-6]. *L. hongkongensis* has been found in up to 60% of the intestines of commonly consumed freshwater fish of the carp family [7,8]. It has also been isolated from drinking water reservoirs and Chinese tiger frogs in Hong Kong and little egrets in Hangzhou [9-11]. Pulsed-field gel electrophoresis and multilocus sequence typing showed that the fish and patient isolates fell into separate clusters, suggesting that some clones could be more virulent or adapted to human [8,12]. These data strongly suggest that this bacterium is a potential diarrheal pathogen that warrants further investigations.

**Figure 1:**
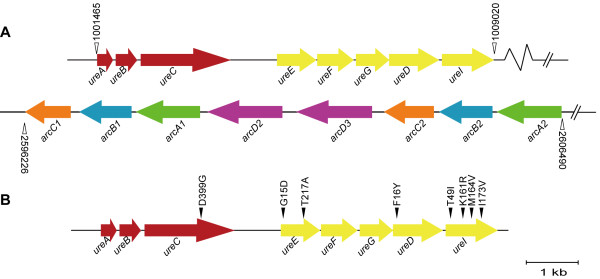
**Genetic organization of urease gene cassette and the two adjacent *arc *gene cassettes. A**, The open vertical triangles represent the locations of the gene cassettes, and the numbering is according to the sequence of the HLHK9 strain. **B**, Schematic illustration showing the differences in the sequences of the urease gene cassettes between *L. hongkongensis* HLHK9 and the naturally urease-negative strain HLHK30. Vertical triangles represent the locations of polymorphic residues, and the numbering is according to the sequence of the HLHK9 strain.

For any gastrointestinal tract pathogen, after transmission through the oral route, the first challenge that the bacterium has to face is the hostile acidic environment of the stomach. When the bacterium invades the intestinal mucosa, it has to survive the attack of submucosal macrophages, which sometimes may be related to its resistance to the acidic environment in endocytic vacuoles. More importantly, for a successful pathogen, the ability of resisting acidic environments is definitely crucial for its survival in different environment and transition from environments to humans. Various gastrointestinal bacteria have developed different mechanisms to overcome this hostile environment and evade host defense. For example, *Helicobacter pylori* and verotoxigenic *Escherichia coli* O157 have developed unique mechanisms to overcome such an acidic environment [13-15]. For *H. pylori*, urease converts urea to carbon dioxide and ammonia and increases the local pH of the bacterium, which is essential for its pathogenesis [16]. During the process of analyzing the *L. hongkongensis* genome, a complete urease cassette, which includes eight open reading frames, encoding three urease structural proteins (UreA, UreB and UreC) and five accessory proteins (UreE, UreF, UreG, UreD and UreI) (Figure 1A), was observed [17]. In addition, two adjacent *arc* gene cassettes, each of them consisting of four genes, *arcA, arcB, arcC* and *arcD* (Figure 1A), were also found [17]. *arcA*, *arcB* and *arcC* encode the three enzymes, arginine deiminase (ADI), ornithine carbamoyltransferase and carbamate kinase, of the ADI pathway; and *arcD* encodes a membrane bound arginine-ornithine antiporter. These enzymes of the ADI pathway convert L-arginine to carbon dioxide, ATP, and ammonia, which increases the pH of the environment and have been shown to be important for survival of various bacteria in acidic environments [18-21]. We hypothesize that the urease cassette and/or the *arc* gene cassettes are important for *L. hongkongensis* to survive in acidic environments and macrophages. In this study, we tested this hypothesis by systematically knocking out genes in the urease cassette and the two *arc* gene cassettes in *L. hongkongensis* and examining their effects in the survival of the single, double and triple knockout mutants in acidic environment *in vitro*, in macrophages and in a mouse model.

## Methods

### Ethics statement

The experimental protocols were approved by the Committee on the Use of Live Animals in Teaching and Research, The University of Hong Kong, in accordance with the Guidelines laid down by the NIH in the USA regarding the care and use of animals for experimental procedures.

### Bacterial strains and growth conditions

The bacterial strains and plasmids used in this study are listed in Table 1. The parental *L. hongkongensis* strain HLHK9, was a clinical isolate from a patient in Hong Kong [3], for which the complete genome has been sequenced [17]. Streptomycin (Sm)-resistant HLHK9 strain was obtained by serial passage of HLHK9 cells on Luria broth (LB) agar with increasing concentrations of Sm, starting at 10 μg/ml, and increased up to 100 μg/ml. Unless stated otherwise, all HLHK9 and its derivate strains used in this study were Sm resistant. HLHK9 and its derivatives were grown in brain heart infusion (BHI) broth or on BHI agar (BHA) plates (BBL, BD) whereas all other *E. coli* strains were grown in LB or on LB agar (LBA) plates (BBL, BD). Media were supplemented with antibiotics (Sigma-Aldrich) when appropriate: ampicillin (Amp) (100 μg/ml), kanamycin (Km) (50 μg/ml), chloramphenicol (Cm) (15 μg/ml), tetracycline (Tet) (12.5 μg/ml) and Sm (100 μg/ml). Growth phase and bacterial cell density were determined by measuring absorbance spectrophotometrically at optical density (OD)_600_.

**Table 1:** Bacterial strains and plasmids used in this study

**Strains or plasmids**	**Characteristics**	**Source or reference**
Strains		
*E. coli* DH5α	F^-^, Ф80d *lacZ*∆M15, ∆(*lacZYA*-*argF*)U169, *endA*1, *recA*1, *hsdR*17(rk^-^, mk^+^) *deoR*, *thi*-1, *supE*44, λ^-^, *gyrA*96(Nal^r^), *relA*1	Invitrogen
*E. coli* SM10(λ pir)	*thi thr leu tonA lacY supE recA*::RP4-2-TC::Mu Km λpir	[23]
*L. hongkongensis* HLHK1 to HLHK30	Thirty human strains isolated from patients with community-acquired gastroenteritis in Hong Kong	[3]
HLHK9	HLHK9 derivative with Sm resistance phenotype, Sm^+^	This study
HLHK9∆*ureA*	HLHK9 derivative with *ureA* deletion, Sm^+^	This study
HLHK9∆*ureC*	HLHK9 derivative with *ureC* deletion, Sm^+^	This study
HLHK9∆*ureD*	HLHK9 derivative with *ureD* deletion, Sm^+^	This study
HLHK9∆*ureE*	HLHK9 derivative with *ureE* deletion, Sm^+^	This study
HLHK9∆*arcA1*	HLHK9 derivative with *arcA1* deletion, Sm^+^	This study
HLHK9∆*arcA2*	HLHK9 derivative with *arcA2* deletion, Sm^+^	This study
HLHK9∆*arcA1*/*arcA2*	HLHK9 derivative with *arcA1* and *arcA2* double deletion, Sm^+^	This study
HLHK9∆*ureA*/*arcA1*/*arcA2*	HLHK9 derivative with *ureA*, *arcA1* and *arcA2* triple deletion, Sm^+^	This study
Plasmids		
pUC19	Cloning vector; *ori lacZ* Amp^r^	Invitrogen
pCVD442	Suicide plasmid; *R6K ori mob RP4 bla sacB*	[25]
pDS132	Suicide plasmid; *R6K ori mob RP4 cat sacB*	[22]
pDS132-*ureA*	pDS132 carrying 5′- and 3′-flanking regions of *ureA* for mutagenesis of *ureA*	This study

### Construction of non-polar deletion mutant strains

Primers used for deletion mutagenesis are listed in Table 2. To generate unmarked, non-polar deletion of *ureA*, suicide plasmid pDS132 was used for constructing in-frame deletion mutants by homologous recombination [22]. 5′- and 3′-flanking regions of *ureA* were amplified by PCR from chromosomal DNA of HLHK9, using primers *ureA*-UF/UR and *ureA*-DF/DR, respectively, and the individual PCR products were mixed to generate an in-frame deletion pattern of *ureA* by an overlapping PCR method. The overlapping amplicon containing the in-frame deletion pattern was cloned into pUC19 followed by pDS132, resulting in the final construct of pDS132-*ureA*, which was electro-transformed into pir-positive *E. coli* SM10 λ pir [23]. pDS132-*ureA* was transferred into HLHK9 from transformed SM10 λ pir by bacterial conjugation. Exconjugants having single recombination with suicide vector pDS132 were first selected on BHA with antibiotics (Cm, 15 μg/ml; Sm, 100 μg/ml). After that, the positive enconjugants with single recombination were further cultured and selected on LBA with 10% sucrose (without NaCl and antibiotics) at room temperature to obtain the final double recombinants [24]. Sucrose-resistant colonies were screened by PCR using primers *ureA*-UF/DR and Inner-*ureA*-F/R, which were specific for the deleted sequence. All mutant strains were confirmed by DNA sequencing.

**Table 2:** Primers used in this study

**Primers**	**Sequence**^**a**^	**Restriction site**
Primers for mutagenesis of *ureA*		
*ureA*-UF	5′ GC*TCTAGA*ATCCTTCATGGGCTGT	*Xba*I
*ureA*-UR	5′ GCAGGGAGCGGTGGATAACCTCCATTTG	
*ureA*-DF	5′ TGGAGGTTATCCACCGCTCCCTGCACAC	
*ureA*-DR	5′ C*GCATGC*TTCCTCATCAGATGGAGCAGACG	*Sph*I
Inner-*ureA*-F	5′ TGCATCTCACTCCGCGTGAG	
Inner-*ureA*-R	5′ TACTGGATCGGGGAATGCAC	
Primers for mutagenesis of *ureC*		
*ureC*-UF	5′ TCA*GAGCTC*CAGGTCGAAGCCGTCTTCAC	*Sac*I
*ureC*-UR	5′ GTTTCCGGGTTACCCAGACGGATCTTG	
*ureC*-DF	5′ TCCGTCTGGGTAACCCGGAAACCTTCG	
*ureC*-DR	5′ TC*GTCGAC*CGTTGGCCACGAAGATGTCC	*Sal*I
Inner-*ureC*-F	5′ GTCCGTCCGGAAACCATTGC	
Inner-*ureC*-R	5′ CCTGCGAGGCAAAGGTGATG	
Primers for mutagenesis of *ureD*		
*ureD*-UF	5′ GC*GAGCTC*CAAGACCGCCATCATCGAAG	*Sac*I
*ureD*-UR	5′ GGTACATCAGGTCAAACGCGGCGATGGC	
*ureD*-DF	5′ CGCGTTTGACCTGATGTACCGGGTGGTG	
*ureD*-DR	5′ GC*GTCGAC*ACCAGATACAGCCACATCAG	*Sal*I
Inner-*ureD*-F	5′ TACAGCAGGCGCTGTACTGG	
Inner-*ureD*-R	5′ GCAGCAGCACGTTGGCAAAG	
Primers for mutagenesis of *ureE*		
*ureE*-UF	5′ CG*TCTAGA*GGAGCCATGTTCCGCGAAT	*Xba*I
*ureE*-UR	5′ GCATGGTGATGCAGGGCAATCTCCACT	
*ureE*-DF	5′ AGATTGCCCTGCATCACCATGCGGAAG	
*ureE*-DR	5′ T*GCATGC*CATCATCGAGGCCAGTCC	*Sph*I
Inner-*ureE*-F	5′ TGGCCAGCATCACGCTCAAG	
Inner-*ureE*-R	5′ TGCAGGTGCTGGTGGGTATG	
Primers for mutagenesis of *arcA1*		
LPW14961 (*arcA1*-UF)	5′ CCG*CTCGAG*TGGATGATCACGGTCAAG	*Xho*I
LPW14962 (*arcA1*-UR)	5′ GTATTGCGGTCCTGTTCAACCCAGATCAC	
LPW14963 (*arcA1*-DF)	5′ GGTTGAACAGGACCGCAATACCTATACC	
LPW14964 (*arcA1*-DR)	5′ CTAG*TCTAGA*TAGCGGGCCAGCTCTTCG	*Xba*I
LPW16076^b^ (Inner-*arcA1*-F)	5′ ACATGCTGACCAGGGTTTG	
LPW16077^b^ (Inner-*arcA1*-R)	5′ AAAGGCTTGTCGTGCCGTTC	
Primers for mutagenesis of *arcA2*		
LPW14965 (*arcA2*-UF)	5′ CCG*CTCGAG*GATTTATTCGCCGGAAAC	*Xho*I
LPW14966 (*arcA2*-UR)	5′ ACACCGCGATCGTAGTCGTGGTCCTTCTG	
LPW14967 (*arcA2*-DF)	5′ CACGACTACGATCGCGGTGTTGAAGTG	
LPW14968 (*arcA2*-DR)	5′ TGC*TCTAGA*GTACATGCGGCCCAGAAC	*Xba*I
LPW16078^c^ (Inner-*arcA2*-F)	5′ ATCGCAAGGTGTGCGCCAAC	
LPW16079^c^ (Inner-*arcA2*-R)	5′ AGCGATTCACGCACCACTTC	
Primers for real-time qPCR		
LPW21629 (*arcA1*-F)	5′GTCACCCTCAATCCGATG	
LPW21630 (*arcA1*-R)	5′CACCACACCTTCACCTTG	
LPW21631 (*arcA2*-F)	5′CCGAAGTGGTGCGTGAATC	
LPW21632 (*arcA2*-R)	5′TCTTGTTGGTGTAGGTGTTGC	
LPW21635 (*rpoB*-F)	5′GTGCTGTTCGTCAATGAG	
LPW21636 (*rpoB*-R)	5′TAGGTCGTAGGATTCTTCG	
LPW22260 (*ureA*-F)	5′ATCTATTGCCTCGCCGAAGTG	
LPW22261 (*ureA*-R)	5′AGTGCTGCCGCCGAAATC	

^a^Restriction sites in the primer sequences are in italic.

^b^Specific primers for amplification of *arcA1.*

^c^Specific primers for amplification of *arcA2.*

Similarly, non-polar deletion of the *ureC*, *ureD* and *ureE* were constructed respectively as described above (Table 2). Instead of using suicide plasmid pDS132, *arcA1*, *arcA2*, *arcA1/arcA2* double mutant and *ureA/arcA1/arcA2* triple mutant strains were constructed using suicide plasmid pCVD442 [25], and Amp was used as the selection marker.

### Qualitative analysis of urease enzyme activity

Thirty human strains, including HLHK9, and mutant strains HLHK9∆*ureA*, HLHK9∆*ureC*, HLHK9∆*ureD* and HLHK9∆*ureE*, were grown at 37°C overnight. Bacterial cultures were diluted 1:50 in BHI containing Sm and further cultured at 37°C with shaking, until early-exponential phase (about 0.6 at OD_600_). One hundred microliter of bacterial cultures was used to inoculate 2 ml urease test broth [26]. The mixtures were incubated at 37°C without shaking. The color change in urease test broth was monitored at 4, 8, 24 and 48 h with the uninoculated urease test as negative control [27].

### Qualitative analysis of ADI activity

A chemical colorimetric method, based on the production of L-citrulline from L-arginine, was used to measure ADI activity of whole-cell lysates of 30 human strains, including HLHK9, and mutant strains HLHK9∆*arcA1*, HLHK9∆*arcA2* and HLHK9∆*arcA1/arcA2*[28,29]. Briefly, 10 ml overnight culture of test strains were re-suspended in 2 ml extraction solution (2% Triton X-100, 1% SDS, 100 mM NaCl, 10 mM Tris pH 8, 1 mM EDTA) and lysed by glass beads (Sigma-Aldrich). One milliliter of supernatants were mixed with 0.4 ml of 100 mM potassium phosphate buffer (containing 10 mM L-arginine) and incubated at 37°C for 1 h. Afterwards, 250 μl of 1:3 (vol/vol) mixture of 95% H_2_SO_4_ and 85% H_3_PO_4_, and 250 μl of 3% diacetylmonooxime solution were added into the samples, followed by boiling for 15 min. Citrulline standard and the uninoculated reagents were used as positive and blank controls, respectively. The development of an orange color was monitored among the tested strains.

### *In vitro* susceptibility of *L. hongkongensis* to acid pH

One hundred microliter of overnight cultures of HLHK9 and derivative mutant strains were inoculated into 5 ml of fresh BHI respectively and grown to exponential phase (OD_600_ 0.6 to 0.8), washed with sterile water, and harvested by centrifugation. The pH of the phosphate buffered saline (PBS, Sigma-Aldrich) was adjusted to 2, 3, 4, 5 and 6 by adding 1 N HCl in the presence or absence of 50 mM urea (for HLHK9, HLHK9∆*ureA*, HLHK9∆*ureC*, HLHK9∆*ureD*, HLHK9∆*ureE* and HLHK9∆*ureA/arcA1/arcA2*) and 50 mM arginine (for HLHK9, HLHK9∆*arcA1*, HLHK9∆*arcA2,* HLHK9∆*arcA1/arcA2* and HLHK9∆*ureA/arcA1/arcA2*). About 10^8^ colony-forming units (CFUs) per ml of bacterial cells were resuspended in PBS of pH 2 to 6 respectively and incubated at 37°C for 1 h. Furthermore, survival of HLHK9, HLHK9∆*ureA*, HLHK9∆*arcA1/arcA2* and HLHK9∆*ureA/arcA1/arcA2* were also monitored at pH 4 after 3 and 5 h incubation respectively. Following incubation, bacterial cells were washed three times in PBS (pH 7.4), and serial dilutions of each culture were spread in duplicate on BHA to determine the number of viable cells [20,30]. The experiments were performed in triplicate from three independent experiments.

### Intracellular survival assays in J774 macrophages

J774 macrophages (Sigma-Aldrich) were grown in DMEM (Gibco) supplemented with 10% fetal bovine serum (FBS, Sigma-Aldrich) at 37°C in an atmosphere of 5% CO_2_. Infection assays were performed as described previously [31,32]. J774 macrophages were seeded to 24-well tissue culture plates at 4 × 10^5^ cells per well and incubated at 37°C with 5% CO_2_ for 24 h before infection. Log-phase bacterial cultures (OD_600_ of 0.6 to 0.7) of the wild type *L. hongkongensis* HLHK9 and mutants were washed twice with sterile phosphate-buffered saline (PBS) and resuspended in antibiotic-free media. Infection was carried out by inoculating 1 × 10^7^ bacterial cells to each well at a multiplicity of infection of about 10:1 and incubated at 37°C for 1 h to allow adhesion and invasion to occur. After that, the culture supernatants were aspirated and the cells were washed three times with sterile PBS. Gentamicin (Sigma-Aldrich) was then added to each well at a concentration of 100 μg/ml and incubated at 37°C for 1 h to kill the extracellular bacteria followed by washing with sterile PBS and replacing the medium with serum-free DMEM containing 25 μg/ml of gentamicin. After 2 and 8 h post-infection, macrophages were lysed with 1% Triton X-100 (Sigma-Aldrich) for CFUs counts. The CFUs recovered from cell lysates after 2 h of phagocytosis were considered as the initial inocula and were used as the baseline values for intracellular survival analysis. CFUs recovered at 8 h were used to calculate the recovery rate of bacterial cells in macrophages. Experiments were repeated in triplicate to calculate the mean of intracellular survival of bacteria.

### RNA isolation and real-time quantitative RT-PCR

At 2 h and 8 h post infection, the macrophage monolayers were washed with PBS and lysed with 1% Triton X-100 (Sigma-Aldrich). Total RNA was then extracted respectively using RNeasy Mini kit (Qiagen), followed by treating with RNase-free DNase I (Roche) at 37°C for 20 min. Reverse transcription was performed using the SuperScript III kit (Invitrogen). Real-time RT-PCR assay was performed in ABI7900HT Fast Real Time PCR machine (Applied Biosystems) with FastStart DNA Master SYBR Green I Mix reagent kit (Roche), as described by the manufacturer. The sequences of the primers used in the quantitative reverse transcription-PCR (qRT-PCR) were listed in Table 2. The mRNA levels of *arcA1* and *arcA2* and *ureA* genes were measured by quantitation of cDNA and the calculated threshold cycle (CT) corresponding to the target gene was calculated as 2^(*Ct*Target - *Ct*Reference)^ and normalized to that of *rpoB* gene [33].

### Survival of *L. hongkongensis* in mouse model

One hundred microliters of overnight cultures of HLHK9 and mutant strains HLHK9∆*ureA*, HLHK9∆*arcA1/arcA2* and HLHK9∆*ureA/arcA1/arcA2* were inoculated into 5 ml of fresh BHI respectively and grown to exponential phase (OD_600_ 0.6 to 0.8). The bacteria were harvested by centrifugation at 5,000 g for 15 min and resuspended in PBS to about 10^9^ CFUs/ml. Five hundred microliters of bacterial suspension were orally inoculated to groups (n = 5) of 6- to 8-week-old female BALB/c mice which were starved for 6 h previously. Mice were sacrificed 120 min after inoculation and the terminal ileum were removed aseptically and homogenized in 5 ml PBS. Serial dilutions of the homogenates were plated in duplicate on BHA with Sm (100 μg/ml) to determine the number of viable cells [30]. The data were collected from three independent experiments.

### PCR amplification and DNA sequencing of *arcA1* and *arcA2*

Extracted DNA from the 30 *L. hongkongensis* human strains previously isolated from stool specimens of patients with community-acquired gastroenteritis [3], was used as template for amplification of *arcA1* and *arcA2* genes, using specific primers LPW16076/16077 and LPW16078/16079, respectively. The PCR mixture (25 μl) contained *L. hongkongensis* DNA, 1× PCR buffer II, 2.0 mM MgCl_2_, 200 μM of each dNTPs and 1.0 unit AmpliTaq Gold DNA polymerase (Applied Biosystems). All samples underwent denaturation at 95°C for 10 min, followed by 40 cycles of 95°C for 1 min, 60°C for 1 min and 72°C for 1 min, with a final extension at 72°C for 10 min in an automated thermal cycler (Applied Biosystems). Five microliters of each amplified product was electrophoresed in 2% (wt/vol) agarose gel and Tris-borate-EDTA buffer, with molecular size marker (GeneRuler 50-bp DNA ladder; Fermentas) in parallel, at 100 volts for 1 h. Five PCR products were randomly selected, gel-purified and sequenced with an ABI Prism 3700 DNA Analyzer (Applied Biosystems), using the PCR primers.

### Statistical analysis

Statistical analyses were performed using Prism 5.01 (GraphPad). CFU counts were logarithmically transformed prior to analysis. Unless stated otherwise, data generated were expressed as mean +/- standard error of the mean (SEM). Statistically significance was calculated using the unpaired student’s *t*-test. p < 0.05 was considered statistically significant (*, p < 0.05; **, p < 0.01; ***, p < 0.001).

## Results

### Examination of *L. hongkongensis* strains for urease activity

**Figure 2:**
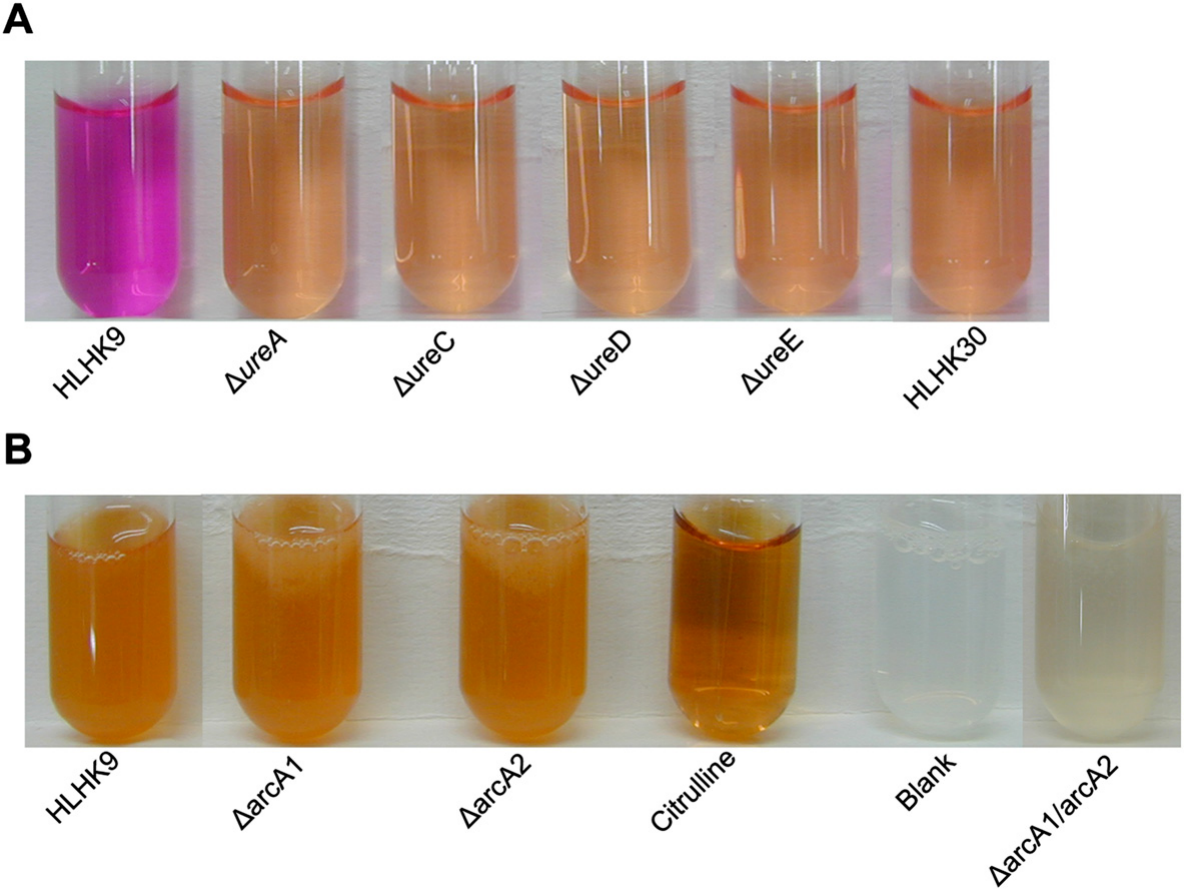
**Examination of *L*. *hongkongensis *strains for urease and ADI activities. A**, A color change from yellow to pink was indicative of positive urease activity. The photo was taken at 8 h post-inoculation. **B**, A color change to orange was indicative of positive ADI activity.

With the exception of native urease-negative *L. hongkongensis* HLHK30, the urease test broth incubated with all human strains, including HLHK9, began to turn pink after 4 h (Figure 2A), and the color became more intense after 24 h of incubation. Similar to the natural urease-negative strain HLHK30, mutant strains HLHK9∆*ureA*, HLHK9∆*ureC*, HLHK9∆*ureD* and HLHK9∆*ureE* elicited no color change after prolonged incubation (Figure 2A). These results indicated that these four urease genes were all essential for the urease enzyme activity.

### Examination of *L. hongkongensis* strains for ADI activity

In the qualitative assay, similar to the positive control (citrulline standard), cellular extracts prepared from all 30 human strains, including wild type *L. hongkongensis* HLHK9, also generated an orange color, confirming that citrulline was being produced (Figure 2B). Cell extracts from both single knockout mutant strains, HLHK9∆*arcA1* and HLHK9∆*arcA2*, also yielded an orange color, whereas deletion of both *arcA1* and *arcA2* abolished the ADI activity (Figure 2B). These results showed that both the *arcA1* and *arcA2* genes encode functional ADI enzymes, which could complement the functions of each other.

### *In vitro* susceptibility of urease-negative mutants to acid

**Figure 3:**
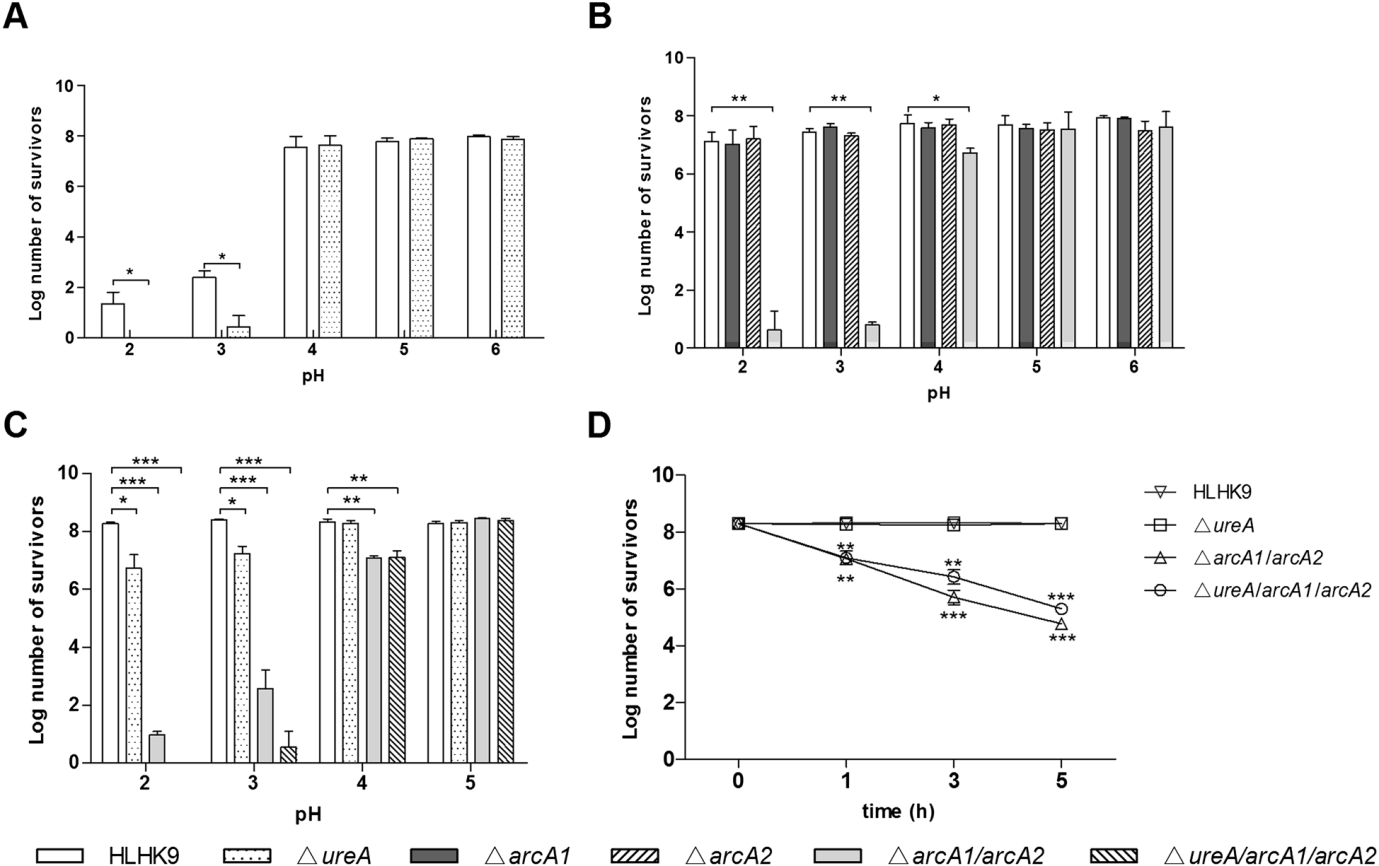
**Survival of wild type *L. hongkongensis *HLHK9 and derivative mutants under acidic conditions.** Survivors were enumerated by plating serial dilutions on BHA plates. Error bars represent means ± SEM of three independent experiments. An asterisk indicates a significant difference (*, p < 0.05; **, p < 0.01; ***, p < 0.001). **A**, Survival of HLHK9 and HLHK9∆*ureA* in the presence of 50 mM urea. **B**, Survival of HLHK9, HLHK9∆*arcA1*, HLHK9∆*arcA2* and HLHK9∆*arcA1*/*arcA2* in the presence of 50 mM arginine. **C**, Survival of HLHK9, HLHK9∆*ureA*, HLHK9∆*arcA1*/*arcA2* and HLHK9∆*ureA*/*arcA1*/*arcA2* in the presence of 50 mM each of urea and arginine. **D**, Survival of HLHK9, HLHK9∆*ureA*, HLHK9∆*arcA1*/*arcA2* and HLHK9∆*ureA*/*arcA1*/*arcA2* at pH 4, and at the indicated time points, in the presence of 50 mM each of urea and arginine.

HLHK9 and mutant strains HLHK9∆*ureA,* HLHK9∆*ureC,* HLHK9∆*ureD* and HLHK9∆*ureE* were subjected to a range of acidic pHs (from pH 2 to 6) in the presence and absence of 50 mM urea, respectively. Since the four urease mutant strains exhibited similar survival abilities under different acidic conditions, only the viable counts of HLHK9∆*ureA* are shown. In the absence of urea, both the parental and mutant strains survived equally well at pH 4 and above, but all strains did not survive at pH 2 and 3 (data not shown). In the presence of urea, there were no significant difference in the survival levels of HLHK9 and urease mutant strains after incubation at pH 5 and 6 for 1 h, with viable counts of all strains declining slightly at pH 4 (Figure 3A). When the pH was further decreased to pH 2 and 3, the survival counts of HLHK9 reduced about 6-log, and the mutant strain could barely be recovered (p < 0.05) (Figure 3A). These demonstrated that the urease system has a contribution to the survival of *L. hongkongensis* at pH 3 and below.

### *In vitro* susceptibility of ADI-negative mutants to acid

To study the role of the two *arc* loci of *L. hongkongensis* under acidic conditions, wild type *L. hongkongensis* HLHK9, HLHK9∆*arcA1*, HLHK9∆*arcA2*, HLHK9∆*arcA1/arcA2* were exposed to different acidic pHs (pH 2 to 6) in the presence and absence of 50 mM of L-arginine, respectively. In the absence of L-arginine, survival of the three mutants were similar to that of HLHK9 at ≥pH 4, and they became susceptible at ≤pH 3 (data not shown). In the presence of L-arginine, wild type *L. hongkongensis* HLHK9, HLHK9∆*arcA1* and HLHK9∆*arcA2* survived well under all tested pHs, suggesting that the two copies of the *arcA* gene performed complementary functions in *L. hongkongensis* (Figure 3B). On the other hand, the survival of HLHK9∆*arcA1/arcA2* decreased about 2-log at pH 4 (p < 0.05) and it was barely recovered at pH 2 and 3 (p < 0.01) (Figure 3B). This indicated that the ADI pathway played a crucial role in the survival of *L. hongkongensis* under acidic conditions.

### *In vitro* susceptibility of urease- and ADI-negative triple knockout mutant to acid

Given the above results that both the urease and ADI pathway contribute towards the overall acid tolerance of *L. hongkongensis*, we constructed a triple knockout mutant strain HLHK9∆*ureA*/*arcA1/arcA2* and compared its survival abilities with HLHK9, HLHK9∆*ureA* and HLHK9∆*arcA1/arcA2* under different acidic conditions in the presence of 50 mM each of L-arginine and urea. The parental and mutant strains displayed similar susceptibilities at pH 5 (Figure 3C). At pH 4, the survival count of HLHK9∆*ureA* was similar to that of HLHK9 but there was about 2-log reduction in that of HLHK9∆*arcA1/arcA2* (p < 0.01) and HLHK9∆*ureA*/*arcA1/arcA2* (p < 0.01) (Figure 3C), and the reduction trend became more pronounced after 3 and 5 h incubation (Figure 3D). At pH 2 and 3, the survival counts of HLHK9∆*ureA* started to decrease (*P <*0.05), whereas there were dramatic decreases in the survival counts of HLHK9∆*arcA1/arcA2* (p < 0.001) and triple knockout mutant HLHK9∆*ureA/arcA1/arcA2* strains, which were almost completely killed (p < 0.001) (Figure 3C). These showed that the ADI pathway of *L. hongkongensis* played a more important role than the urease in resisting acidic environments.

### Intracellular survival in J774 macrophages and mRNA expression level analyses

**Figure 4:**
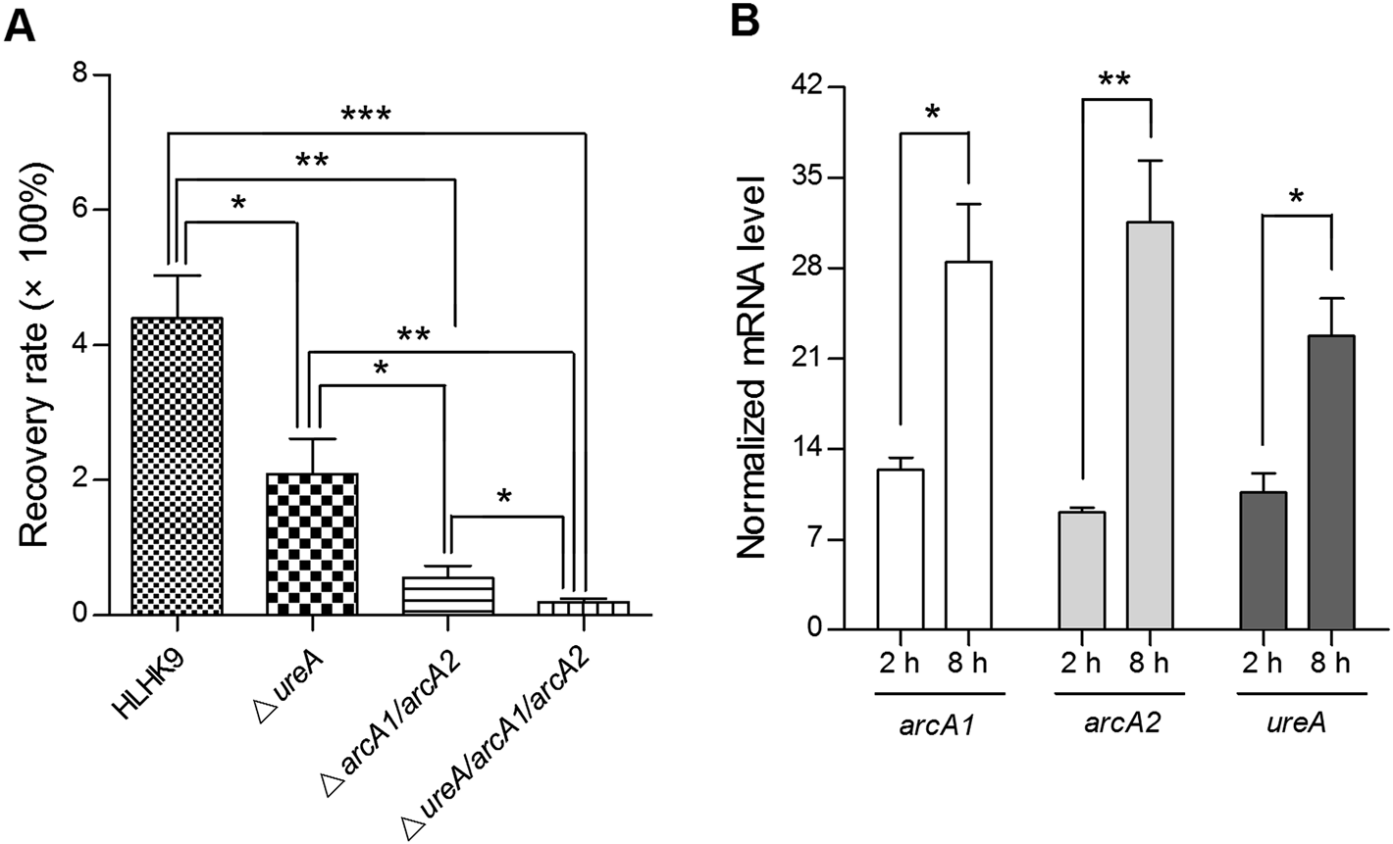
**Intracellular survival assays in J774 macrophages. A**, Recovery rates of wild type *L. hongkongensis* HLHK9, HLHK9∆*ureA*, HLHK9∆*arcA1/arcA2* and HLHK9∆*ureA/arcA1/arcA2* in J774 macrophages. **B**, Expression level of ADI genes (*arcA1* and *arcA2*) and *ureA* gene of HLHK9 in macrophages. Error bars represent means ± SEM of three independent experiments. An asterisk indicates a significant difference (*, p < 0.05; **, p < 0.01; ***, p < 0.001).

Survival of wild type *L. hongkongensis* HLHK9, HLHK9∆*ureA*, HLHK9∆*arcA1/arcA2* and HLHK9∆*ureA*/*arcA1/arcA2* in J774 macrophages were shown in Figure 4A. Survival of HLHK9∆*ureA/arcA1/arcA2* and HLHK9∆*arcA1/arcA2* in macrophages were markedly decreased (p < 0.001 and p < 0.01 respectively) but that of HLHK9∆*ureA* was slightly decreased (p < 0.05), compared to wild type *L. hongkongensis* HLHK9. The decrease of survival was more prominent in HLHK9∆*ureA*/*arcA1/arcA2,* compared to HLHK9∆*arcA1/arcA2* (p < 0.05) and HLHK9∆*ureA* (p < 0.01); and in HLHK9∆*arcA1/arcA2,* compared to HLHK9∆*ureA* (p < 0.05). Given the above results, we further investigated the expression level of ADI genes (*arcA1* and *arcA2*) and *ureA* gene of wild type *L. hongkongensis* HLHK9 survived in macrophages using real-time quantitative RT-PCR assay. At 8 h post infection, the mRNA levels of *arcA1*, *arcA2* and *ureA* genes were markedly increased compared to those at 2 h post infection (p < 0.05, p < 0.01 and p < 0.05 respectively) (Figure 4B).

### Survival of *L. hongkongensis* strains in BALB/c mice

**Figure 5:**
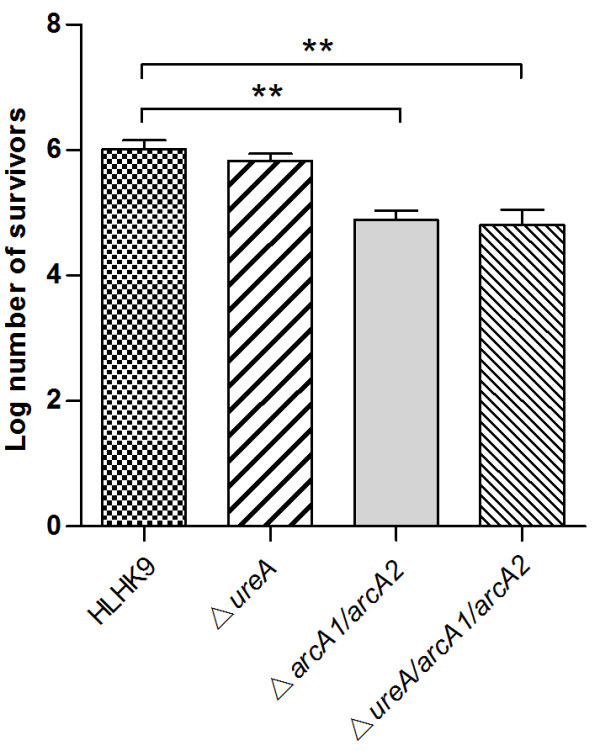
**Survival of wild type *L. hongkongensis *HLHK9 and derivative mutants using a mouse model.** Error bars represent means ± SEM of three independent experiments. An asterisk indicates a significant difference (**, p < 0.01).

To further investigate the role of urease and ADI pathway in acid tolerance of *L. hongkongensis*, we compared the survival ability of HLHK9, mutant strains HLHK9∆*ureA*, HLHK9∆*arcA1/arcA2* and HLHK9∆*ureA*/*arcA1/arcA2* after transit through the stomach of mice. Using this mouse model, HLHK9∆*ureA* exhibited similar survival abilities as HLHK9 (Figure 5). In contrast, the viable counts of HLHK9∆*arcA1/arcA2* and HLHK9∆*ureA/arcA1/arcA2* were reduced by 1.2-log and 1.3-log respectively, compared to that of HLHK9 (p < 0.01) (Figure 5). This also indicated that the ADI pathway played a more significant role than urease in the survival of *L. hongkongensis* under the acidic conditions encountered during passage through the mouse gastric environment.

### PCR amplification and DNA sequencing of *arcA1* and *arcA2*

A specific 739-bp fragment of *arcA1* and a specific 712-bp fragment of *arcA2* of *L. hongkongensis* were amplified from the DNA extracts of all 30 human strains, indicating that both *arcA1* and *arcA2* were present in all 30 human strains. DNA sequencing of the PCR products from five randomly selected *L. hongkongensis* strains confirmed that the amplified products were *arcA1* and *arcA2* respectively. Sequence analyses showed that there were 1 to 5 nucleotide differences and one amino acid difference between the 739-bp fragments and the deduced amino acid sequences of the *arcA1* genes from these five selected strains and the corresponding region of HLHK9. Similarly, there were 1 to 4 nucleotide differences but no amino acid difference between the 712-bp fragments of the *arcA2* genes from these five strains and the corresponding region of HLHK9. Sequence analysis also revealed that most of the conserved residues were present in the partial fragments of *arcA1* and *arcA2*, compared to ADI sequences of other bacteria.

## Discussion

We showed that the *arc* gene cassettes are more important than the urease gene cassette for acid resistance and survival in macrophages in *L. hongkongensis*. Although both urease and *arc* gene cassettes have previously been reported to play roles in acid resistance in bacteria, urease function appears to be more important in gastrointestinal tract bacteria such as *H. pylori*, *Yersinia enterocolitica* and *Klebsiella pneumoniae*[16,30,34]. In fact, the mechanisms of acid resistance are similar in both reactions, which result in production of ammonia, thereby increasing the pH of the immediate environment of the bacterium. As for survival in macrophages, ADI pathway has been shown to contribute to survival in macrophages in *Salmonella* Typhimurium [32], but not in *Listeria monocytogenes*[29]; and urease has been shown to contribute to survival in macrophages in *H. pylori*[35], but not in *Brucella suis* and *Brucella abortus*[30,36]. To the best of our knowledge, the present study is the first to compare the relative importance of these two acid resistance and intracellular survival mechanisms using *in vitro* and *in vivo* models, although these two gene cassettes are present in many gastrointestinal tract bacteria, such as *Y. enterocolitica* and *Enterobacter cloacae*. By constructing a series of urease knockout mutants, we found that both structural and accessory genes in the urease gene cassette are crucial for the urease activity; which is in line with previous studies performed in other bacterial species [15,30,37]. Contrary to our initial hypothesis, we observed only a small reduction in survival abilities of the urease knockout mutants in acidic media (pH 2 and 3) and macrophages as well as during gastric passage in the mouse model. This is consistent with our previous recovery of a strain of urease-negative *L. hongkongensis* (HLHK30) from an 84-year old male with gastroenteritis. Sequencing of the urease cassette of HLHK30 showed that all eight of the component genes were present with no deletions or frame shift mutations; although there were a number of polymorphic sites that resulted in amino acid changes compared to gene homologues present in HLHK9 (Figure 1B). On the other hand, the ADI-deficient mutant HLHK9∆*arcA1/arcA2* showed marked reduction in survival abilities in acidic media and macrophages as well as in the mouse model, indicating that *arc* gene cassettes play a more important role than urease gene cassettes for acid resistance in *L. hongkongensis*. In fact, the survival abilities of the triple knockout mutant strain HLHK9∆*ureA*/*arcA1/arcA2* were only marginally lower than those of the ADI-deficient double mutant strain HLHK9∆*arcA1/arcA2* in acidic media and macrophages, and both mutant strains had equivalent survival abilities in the mouse model, which further supports the conclusion that ADI play a more important role.

The gene duplication of the *arc* gene cassettes could be a result of their functional importance in *L. hongkongensis*. One of the important mechanisms of virulence evolution in bacteria and fungi is gene duplication [38-40]. *L. hongkongensis* is the only bacterium known to possess two adjacent *arc* gene cassettes. The *L. hongkongensis* mutant strain containing deletions of the *arcA* genes in both *arc* cassettes exhibited a marked reduction in survival abilities compared to the mutant strains containing single deletion of either one of the two *arcA* genes, indicating that both *arc* gene cassettes are functional and contribute to acid resistance. Phylogenetic analysis showed that the two copies of *arc* in *L. hongkongensis* are clustered in all the four trees constructed using *arcA*, *arcB arcC* and *arcD*[41]. This strongly suggests that the two *arc* gene cassettes result from a gene cassette duplication event. Interestingly, in our previous study on differential gene expression in *L. hongkongensis* at different temperatures, it was observed that the two copies of *argB*, encoding two isoenzymes of *N*-acetyl-L-glutamate kinase from the arginine biosynthesis pathway, which have distinct biochemical properties, are also clustered phylogenetically [17]. This indicates that these two copies of *argB* probably also arose as a result of gene duplication. Subsequent evolution enabled the two copies of *argB* to adapt to different temperatures and habitats. These coincidental findings of gene duplication in two different pathways of arginine metabolism, enabling the bacterium to better adapt to different environmental conditions, *argB* for temperature adaptation and *arc* gene cassette for acid resistance, is intriguing.

The present study further strengthened the feasibility of using a conjugation mediated gene deletion system based on a suicide vector in the *Neisseriaceae* family of β-proteobacteria, a strategy which has been widely used in γ-proteobacteria, such as *E. coli*, *Salmonella* Typhimurium and *Vibrio cholera*[22,42,43]. In our previous studies on plasmid transformation and gene expression system in *L. hongkongensis*, we observed that plasmids commonly used for expression systems in *E. coli* did not replicate in *L. hongkongensis*[44]. Therefore, an *E. coli- L. hongkongensis* shuttle vector, based on a *L. hongkongensis* plasmid backbone and origin of replication, was constructed [44]. In our subsequent gene deletion experiments in *L. hongkongensis*, we used a pBK-CMV plasmid that harbored 1000 bp of genomic upstream and downstream of the target gene, but lacked the target gene, which was transformed into *L. hongkongensis*. This gene deletion system was successfully used to delete several *L. hongkongensis* genes, such as the *flgG* flagellar gene. However attempts to delete the *ureA, ureB, ureC* and *ureI* genes were all unsuccessful (unpublished data). Therefore, the present gene deletion system, which was first used in *E. coli*[42], and also recently used in *Chromobacterium violaceum*, another pathogenic bacterium of the *Neisseriaceae* family [45], was used for knocking-out genes from the urease and *arc* gene cassettes. Further experiments will elucidate whether this gene deletion system is also useful for knocking out genes in other important bacteria of the *Neisseriaceae* family, such as the *Neisseria gonorrhoeae* and *Neisseria meningitidis*.

## Conclusions

ADI pathway is far more important than urease for acid resistance and intracellular survival in *L. hongkongensis*. The gene duplication of the *arc* gene cassettes could be a result of their functional importance in *L. hongkongensis*.

## Abbreviations

Bp: Base pairs; DNA: Deoxyribonucleic acid; μg: Microgram; ml: Milliliter; μl: Microliter; M: Molar; min: Minutes; PCR: Polymerase chain reaction; h: Hour; DMEM: Dulbecco's modified eagle medium; EDTA: Ethylenediaminetetraacetic acid; SDS: Sodium dodecyl sulfate; NaCl: Sodium chloride.

## Competing interests

The authors declare that they have no competing interests.

## Authors’ contributions

PCYW and JLLT conceived the study. PCYW, JLLT and LX wrote the manuscript; PCYW, JLLT, LX and SKPL participated in the design of the study; LX and JLLT performed the experiments. LX, JLLT and PCYW analyzed the data; RMW, BK and SKPL corrected the manuscript; all authors read and approved the final manuscript.
